# Quality of life of patients one year after breast-conserving surgery versus modified radical mastectomy for early breast cancer: a Kenyan tertiary hospital five-year review

**DOI:** 10.11604/pamj.2023.46.69.39151

**Published:** 2023-10-25

**Authors:** Andrew Senoga, Ronald Wasike, Sitna Ali Mwanzi, Miriam Mutebi

**Affiliations:** 1 Department of Surgery, Aga Khan University Hospital, Nairobi, Kenya,; 2 Department of Surgery, Masinde Muliro University Medical School, Kakamega, Kenya,; 3 Medical Oncology, Cancer Treatment Center, Kenyatta National Hospital, Nairobi, Kenya

**Keywords:** Quality of life, early breast cancer, modified radical mastectomy, breast-conserving surgery

## Abstract

**Introduction:**

Breast conserving surgery (BCS) followed by radiotherapy (BCT) and modified radical mastectomy (MRM) are the most common surgical techniques utilized in treatment of early breast cancer (EBC) with similar overall survival and recurrence rates. Western literature suggests that these treatments impact the quality of life (QOL) of patients variably. There are no comparison studies on these treatments as per patient’s QOL in East Africa. The objectives were to compare the QOL of patients with EBC at least one year after BCT or MRM and assess the factors that affect this QOL.

**Methods:**

this was a cross-sectional study conducted at Aga Khan University Hospital-Nairobi (AKUHN). Eligible female patients with EBC who had undergone either BCT or MRM between January 2013 and December 2018 were invited to fill out European Organization for the Treatment and Research of Cancer Quality of Life Questionnaire (EORTC-QLQ-C30). Data on participant demographics and clinical information was also obtained. Average scores for each aspect of QOL were obtained and overall means for each surgical treatment were compared. Linear regression was done to assess the factors that affected this QOL

**Results:**

forty-two patients had BCS/BCT and 39 had MRM. Patients who had undergone BCS/BCT had a better overall QOL than those who had undergone MRM (p=0.0149). Multivariate analysis revealed that five years from time of surgery, level of education and diabetes mellitus significantly (p<0.05) affected the QOL of these patients.

**Conclusion:**

after one year from surgery for EBC, patients who had undergone BCS/BCT had a better QOL as compared to MRM.

## Introduction

As per GLOBOCAN 2020, Kenya registered 6799 new cases of breast cancer as a leading malignancy for all sexes. This contributed a percentage of 16.1% of all new cancer cases that year. Breast cancer also had the highest incidence rate at 41.0 per 100000 (age-standardized rate) and the third highest mortality rate of 19.4 per 100000 just below prostate and cervical cancer in that order [1].

Breast cancer in Africa; Kenya inclusive, occurs in a younger female population in comparison to their Western counterparts with a mean age of 51.9 years compared to 10 years older in Western populations [2,3]. Thus, breast cancer in Africa typically affects women in the 25-54 years´ age bracket, which is the prime working age group and anecdotally the most productive social-economically. The disease is frequently diagnosed at advanced stages and has a more aggressive course as compared to Western populations [3,4]. However, recent studies in Kenya revealed that 41.1% to 32.5% of patients were diagnosed with early breast cancer (EBC) [5,6]. The proportion of patients presenting with EBC is bound to increase with ongoing awareness campaigns and widespread screening.

Curative surgical management of EBC is by either breast conservation surgery (BCS) or modified radical mastectomy (MRM). Breast conservation therapy (BCT) involves cosmetically acceptable wide excision of the tumor with a concentric margin of surrounding health tissue (BCS) plus a sentinel lymph node biopsy or axillary clearance. This is usually followed up with radiotherapy to prevent recurrence. On the other hand, MRM involves excision of the breast with dissection of the associated draining lymph nodes up to anatomical level II also for curative purposes. Adjuvant hormonal, targeted therapy, chemotherapy, and /or radiotherapy may be given to clear micrometastases, prevent recurrence, and improve survival rates regardless of the surgical intervention [7]. BCT has been found to be equivalent to MRM for the management of EBC in terms of disease-free survival and recurrences. Most of these studies are from developed countries [8-14]. Developing countries are starting to embrace BCS/BCT with progressive technological developments and the availability of surgical and other professional expertise. Breast reconstructive surgery after mastectomy can be done as an immediate or delayed procedure. However, this is done infrequently in sub-Saharan Africa due to out-of-pocket costs to patients as this is still viewed as largely cosmetic or due to available skills. Health-related quality of life (HR-QOL) as a patient-reported outcome [15], which is also stated as QOL in this document; is a person´s perception of his or her mental, or physical and social health status as per situation or time. This QOL has been assessed for EBC patients after BCS and MRM in other regions of the world and has been found to vary from place to place with different significant factors being responsible [16-18]. Thus surgical management of EBC has been seen to affect all domains of QOL variably with most of the causation arising from surgical complications, the attendant side effects of the subsequent therapy, and existing co-morbidities of the patients [19].

There are no studies on the QOL of patients after BCT versus MRM for EBC and associated factors in East Africa yet this impacts on the holistic management of EBC in these patients. Thus this study´s aims were to compare the quality of life of female patients one year after BCT versus MRM for EBC and assess the factors that affect this QOL.

**Null hypothesis**: there is no difference in QOL between patients after breast conservation surgery and those after modified radical mastectomy for EBC.

The primary objective was to compare the QOL of female patients at least one year after BCT versus MRM for EBC. The secondary one was to assess the factors that affect this QOL of patients one year after surgery for EBC.

## Methods

**Study design and setting:** a single-center cross-sectional study was conducted at Aga Khan University Hospital-Nairobi (AKUHN) Kenya which is one of the East Africa regional leading hospitals offering specialized breast cancer treatment and host´s one of the oldest dedicated breast centres in the region. It serves patients of all walks of life with the help of the welfare program which also provides financial aid for a selected few. It is also a destination for patients looking for quality health care in East Africa.

**Participants:** between 2013-2018, female patients with EBC who were at least one year after their surgical management (either BCT or MRM) and were over 18 years of age were eligible to participate. Excluded were those with confirmed primary metastatic disease, secondary primary malignancy, and recurrent metastatic disease. A convenience sampling technique was used to get the desired number of participants. Participants were traced from hospital records, contacted, and consented to fill in questionnaires and a data collection sheet during their surgical clinics, oncology clinics, and breast cancer survivors´ meetings.

**Data sources, tools, and variables:** participants were traced from hospital records, contacted, and consented to fill in questionnaires (see Annex 1, Annex 2) and a data collection sheet (see Annex 3). The data collection sheet was used to capture demographic and clinical characteristics data such as their age, marital status, type of surgery that was done, comorbidities, and education level and treatment modality at the time in concert with the clinical records. These were the independent variables. The QOL scores were the dependent variables. We used standard QOL English and Swahili validated questionnaires; that is EORTC QLQ C30 (see Annex 1, Annex 2) to assess participants' QOL. This is a generic QOL questionnaire for all types of cancer patients. It assesses quality of life through 30 domain questions categorized into; functional scales; i.e. physical, emotional, social, role, and cognitive functioning; symptom scale; i.e. loss of appetite, constipation, dyspnea, pain, fatigue, nausea/vomiting, insomnia, and financial difficulties, and global health status/QOL. Scores from each of these scales are converted to a score from 0-100. Higher scores for global health status and functional scale signify better QOL while a higher score for the items under the symptom scale signifies a poor QOL. Clinically important changes in the scores are classified as: a small change was at a score of 10, moderate change at 10-20 and a large change was more than 20 as per Osoba *et al*. [20]. Global health status/QOL score is the domain used as the overall summary score of the quality of life [21].

**Study size:** to assess for a difference between QOL post-BCT and MRM, the sample size was calculated using the formula for comparing two groups´ means of equal numbers as below [22]. We assumed a moderate change of 15% to be a significant change and a standard deviation of 22.7 [21]. The study was powered at 80% with a level of significance of 5%. The calculated participants on each arm were 36.


$$n_{A}=kn_{B}\text{ and }n_{B}=\left(1+\frac{1}{k}\right){\left(\sigma \frac{z_{1-\alpha /2}+z_{1-\beta }}{\mu_{A}-\mu_{B}}\right)}^{2}$$

Where: nA=nB, K=1. Where: nA =number of participants in group A (MRM). nB=number of participants in group B (BCT). δ= standard deviation for global health status =22.7. μA-μB= difference in the observed means=15. Z is the normal deviate. Z (1-α/2); z value for level of significance of 0.05= 1.96. Z (1-β/2); z value for 80% power (1-ß) = 0.84. After substitution; nA=nB= 36 participants. Allowing for a 5% fallout in the participants, each group (A and B) required 37 participants.

**Study bias:** participant bias and research bias we circumvented by the use of a standard verified questionnaire both in English and the commonest native language; Swahili. Research assistants were availed to assist in cases of when participants had difficulties using the questionnaire. Convenience sampling was prone to sampling bias. To reduce this, participants were recruited on varying days, at different clinics (oncology, surgical), and at survivors´ meetings. Others were also identified and called on the telephone in case they were not due for clinic reviews.

**Statistical analysis:** predictors of QOL were described using percentages for categorical variables. Numerical predictors were summarised as means and standard deviations between the two groups and corresponding p-values obtained using student´s T-test and Mann-Whitney U test. Linear regression was carried out using Stata 14 after testing for assumptions. Bivariate linear regression and multivariate linear regression were used. We first checked for the association between independent variables and dependent variables. Variables with p-values less than 0.2 from bivariate analysis and those suggested in the literature were considered for multivariate analysis. Assumptions of multi-collinearity and outliers were then assessed before fitting the multivariate linear regression model. During model building, we assessed for significant interaction terms and confounders. Variables were considered statistically significant if the p-value was <0.05. Approval of this study was by the AKUHN ethics and research committee under Ref: 2019/IERC-61(v2). Confidentiality and anonymity for participants was maintained and data was secured under key and lock.

## Results

### Participants

Two hundred and forty-three (243) patients underwent BCS and 555 had MRM over the five years. A total of 241 patients were eligible to participate in the study. Only 81(33.6%) responded and consented to participate in the study. Almost 84% (68) of the participants were picked up at the review clinics and the rest from the breast cancer survivor's meetings.

### Descriptive data

***Age and socio-demographic:*** the 25-54 years´ age group had the highest number of participants at 52 (64.2%) followed by the 55-64 age group at 16 (19.75%) participants. There was only 1 participant below 24 years of age and 12 participants (14.81%) above the age of 65 years. (Table 1) The majority of the participants were married; i.e. 55(67.9%), 15 (18.52%) were single, and the remainder were either widowed or divorced. Most participants had a tertiary level of education at 54 (66.67%). Those with a primary level of education were 5 (6.17%).

***Clinical characteristics:*** there were comparable numbers of participants in the BCT and MRM groups at 42 (51.85%) and 39 (48.15%) participants respectively. Twenty-three (28.4%) of the participants were at least 1-year post-surgery, 10 (12.35%) were at least 3 years post-surgery and there were 16 (19.5%) participants each at 2, 4 and 5 years after surgery. Hypertension was the most common comorbidity with 20 participants (24.69%). Two participants had diabetes (2.4%) and 6 (6.74%) participants had both hypertension and diabetes. The rest of the 55 (66.67%) participants didn´t report any comorbidity. 69.14% of the participants were not on any form of active treatment for cancer at the time of the study. 13.58% were on chemotherapy/targeted therapy and 17.28% were on endocrine therapy. There was a relatively balanced number of participants in both arms of surgery for each variable category (Table 1).

**Table 1:** demographic and clinical characteristics

Variable	TOTAL = 81		
	**BCS (42)**	**MRM (39)**	**N (%)**
**AGE**			
<25	1	0	1 (1.23)
25-54	30	22	52 (64.20)
55-64	5	11	16 (19.75)
>64	6	6	12 (14.81)
**TIME FROM SUGERY (years)**			
1	13	10	23 (28.40)
2	7	9	16 (19.75)
3	6	4	10 (12.35)
4	7	9	16 (19.97)
5	9	7	16 (19.75)
**COMORBIDITIES**			
Hypertension (HTN)	8	12	20 (24.69)
Diabetes (DM)	1	1	2 (2.4)
HTN / DM	3	3	6 (6.74)
Others	0	0	0 (0)
None	33	20	53 (65.43)
**LEVEL OF EDUCATION**			
Primary	2	3	5 (6.17)
secondary	8	14	22 (27.16)
Tertiary	32	22	54 (66.67)
**MARITAL STATUS**			
Married	30	28	55 (67.9)
Divorced	3	5	8 (9.88)
Single	9	6	15 (18.5)
Widow	1	1	3 (3.70)
**CURRENT TREATMENT**			
None	19	27	56 (69.14)
chemotherapy	5	6	11 (13.58)
endocrine therapy	11	3	14 (17.28)

HTN-hypertension, DM-diabetes mellitus

***Primary outcome***

Overall QOL of patients post BCT vs BCS: global health status/QOL scores in both arms were found to be non-parametric and thus a two-sample Wilcoxon rank-sum test was used. As per this test, BCS significantly offered a better QOL one-year post-surgery as compared to MRM with a z value of 2.434 giving a p-value of 0.0149 (level of significance at less than 5%) and a chance of 0.654 (65.4%) in support of the alternate hypothesis. Therefore, we rejected the null hypothesis.

Comparison of the symptom and functional scores for BCT Vs MRM: on the functional scale in Table 2, the higher the scores, the better the QOL as per that domain. Patients who had BCS were noted to have higher scores with social functioning (p=0.036) and physical functioning (p=0.007) in comparison to the MRM group. On the symptom scale in Table 3 the higher the symptom score, the worse the symptom. All symptoms scored lower in the BCS group implying fewer symptoms in comparison to the MRM group. However, the only statistically significant difference was in fatigue symptoms (p= 0.010).

**Table 2:** comparison of the functional scales on EORTC QLQ - C30 questionnaire

Functional scale	BCS. Mean (SD)	MRM Mean (SD)	p-value
**Physical functioning**	**90.79 (9.07)**	**83.51 (14.09)**	0.007*
Role functioning	89.29 (15.97)	85.96 (19.58)	0.391
Emotional functioning	88.69 (18.75)	80.48 (19.30)	0.064
Cognitive functioning	86.51 (17.74)	77.63 (25.49)	0.052
**Social functioning**	**92.46 (15.27)**	**82.89 (24.66)**	0.036*

SD- Standard Deviation, p-value- Probability value.

**Table 3:** comparison of the symptom scale on the EORTC QLQ - C30 questionnaire

Symptom scale	BCS. Mean (SD)	MRM Mean (SD)	p-value
**Fatigue**	**10.85 (13.55)**	**21.93 (23.74)**	0.010*
Nausea	4.37 (9.06)	7.02 (13.22)	0.323
Pain	14.29 (20.01)	22.81 (23.06)	0.083
Dysnea	1.587 (7.18)	6.14 (17.08)	0.265
Insomnia	14.29 (23.45)	23.68 (30.91)	0.15
Appetite loss	4.76 (17.38)	14.04 (26.43)	0.073
Constipation	6.35 (15.16)	12.28 (23.79)	0.202
Diarrhea	3.17 (9.90)	3.51 (12.94)	0.923
Financial difficulties	28.57 (39.35)	37.72 (37.30)	0.945

***Secondary outcomes***

***Factors that affect QOL patients one-year post-BCS Versus MRM***

***Bivariate analysis:*** bivariate analysis (Table 4) revealed that age, type of surgery, comorbidities and level of education had statistically significant effects on the QOL of patients one year after surgery. The level of significance was at 20% to avoid rejecting a difference that actually exists (type 1 error) during the initial analysis. As regards age, the age group of 55-64 years had their QOL score lowered by a factor of 31.25 for every unit increase in age as compared to the reference age group (0.090). The QOL score was increased by a factor of 21.136 and 25.401 among patients with secondary (p=0.015) and tertiary level (p=0.002) respectively as compared to the primary level of education. Having DM as comorbidity lowered the QOL score by a factor of 46.54 (p<0.2) as compared to having no comorbidity. MRM lowered the QOL score by a factor of 5.983 (p=0.137) as compared to BCS.

**Table 4:** bivariate analysis for factors that affect QOL

Variable		β-Coefficient	P-Value	95% Confidence Interval
Age				
	15 - 24	1	1	1
	25 -54	-21.154	0.238	-56.606 - 14.299
	55 - 64	-31.25	0.090*	-**67.447 - 4.947**
	>65	-17.361	0.347	-53.911 - 19.189
Marital status				
	Single	1	1	1
	Married	-2.979	0.578	-13.603 - 7.644
	Widowed	-5.556	0.633	-28.622 - 17.510
	Divorced	-6.597	0.413	-22.564 - 9.369
Surgery				
	BCS	1	1	1
	MRM	-5.983	0.137*	**-13.914 - 1.948**
Time from surgery				
	1 year	1	1	1
	2 years	6.884	0.246	-4.985 - 18.617
	3 years	0.217	0.975	-13.435 - 13.869
	4 years	3.759	0.525	-7.974 - 15.492
	5 years	-4.57	0.44	-16.308 - 7.159
Comorbidity				
	None	1	1	1
	HTN	-1.958	0.658	-10.734 - 6.818
	DM	-46.541	0.000*	**-70.630 - -22.451**
	HTN/DM	-7.651	0.298	-33.057 - 6,752
Level of education				
	Primary	1	1	1
	Secondary	21.136	0.015*	**4.176 - 38.097**
	Tertiary	25.401	0.002*	**9.399 - 41.404**
Treatment				
	None	1	1	1
	Chemotherapy	-0.122	0.984	-12.029 - 11.785
	Endocrine	6.101	0.264	-4.687 - 16.889

***Multivariate analysis:*** multivariate analysis (Table 5) utilized significant factors from bivariate analysis plus those factors that are known from the literature to affect QOL of patients after surgery. The level of significance was set at 5%. Findings showed that level of education, time from surgery and comorbidities were statistically significant factors that affect the QOL scores. Specifically; DM reduced the QOL of participants by a factor of 54.768 as compared to those patients with no comorbidities (β=-54.578, CI -85.679 - -34.726, p=0.000). Patients at 5 years post-surgery had a lower QOL score by a factor of 12.329 as compared to those at 1-year post-surgery (β= -12.329, CI -24.265 - -2.373, p= 0.022). Being at a higher level of education improved the QOL scores by a factor of 31.85 as compared to those with a primary level of education (ß=31.85, CI 16.913 - 51.139, p=0.000).

**Table 5:** multivariate analysis factors that affect QOL

Variable		β -Coefficients	P-value	95% Confidence Interval
Age				
	15 - 24	1	1	1
	25 -54	-14.134	0.388	-46.656 - 18.387
	55 - 64	-8.149	0.637	-42.463 - 26.164
	>64	-3.103	0.897	-37.599 - 31.392
Marital status				
	Single		1	1
	Married	0.513	0.916	-9.162 - 10.188
	Widowed	-8.47	0.44	-30.242 - 13.302
	Divorced	-0.923	0.897	-15.047 - 13.201
Treatment				
	None	1	1	1
	Chemotherapy	-0.059	0.991	-9.328 - 12.462
	Endocrine	5.682	0.276	-4.225 - 16.881
Time from surgery				
	1 year	1	1	1
	2 years	-3.329	0.544	-14.156 - 8.27
	3 years	-8.116	0.201	-21.769 - 5.028
	4 years	-6.645	0.224	-19.389 - 4.675
	5 years	-12.329	0.022*	**-24.265 - -2.373**
Surgery				
	BCS	1	1	1
	MRM	-4.218	0.245	-11.46 - 3.496
Comorbidity				
	None	1	1	1
	HTN	7.634	0.091	-4.674 - 14.593
	DM	-54.768	0.000*	**-85.679 - -34.726**
	HTN/DM	-10.5	0.135	-28.497 - 0.768
Level of education				
	Primary	1	1	1
	Secondary	28.106	0.001*	**11.718 - 45.837**
	Tertiary	31.857	0.000*	**16.913 - 51.139**

* Level of significance, p<0.05

## Discussion

Curative surgery for EBC in the form of either BCS/BCT or MRM has been seen to have comparable survival and recurrence outcomes [8-11]. QOL assessment in clinical practice is gaining interest as a patient-reported outcome that defines the success of an intervention especially in cancer treatment and care [15]. QOL assessment in patients post BCS vs MRM for EBC has been found to vary for different study populations in the West and some Asian countries [16,23-25]. This is the first study on QOL after breast cancer surgery to be conducted in East Africa. The aim of our study was to compare the QOL of patients after MRM and BCS for EBC and associated factors affecting this QOL. We used the EORTC QLQ C-30 to assess this QOL of patients.

In our study, the age group with the highest percentage of patients with EBC was 25-54 years which is also the prime working age group as per Kenya age groupings [26]. This is a reflection of the general finding of the age distribution of breast cancer patients at diagnosis in Kenya. Our finding is similar to Makanga *et al*. in this same hospital where the median age at diagnosis of breast cancer was 50 years [6]. Fregene *et al*. in a study of African American women also found that these women had a younger age at diagnosis and a more severe disease compared to their Western counterparts [3]. This was attributed to the genetic makeup of the African population and not entirely the environmental risk factors and social-economic disparities [2,3,6]. Over the 5-year period, out of 798 of the breast surgeries done for breast cancer in AKUHN, 248 (30.45%) were breast-conserving surgery. There is no robust data on BCS rates in Africa; however, Vanderpuye V *et al*. reported the mastectomy rate to be almost 90% in Africa while the mastectomy to BCS rates in Europe were 30% and 70% respectively [27]. The observed relatively high rate of BCS in our study as compared to the rest of Africa is explained by the availability of the surgical and pathology expertise to support the procedure and radiotherapy services at this unit which is a vital component in BCS. This is not readily available in many parts of sub-Saharan Africa.

### Breast conservation surgery versus modified radical mastectomy

In this study, the QOL of the participants was summarised by the global health status score from the EORTC QLQ C30 questionnaire. Other parameters on the functional scale and symptom scale were also analysed. Patients one-year post-BCS had a statistically significant better QOL score as compared to their counterparts in the MRM group (p=0.0149) as per the Wilcoxon rank-sum test. The BCS group had better physical functioning (p=0.007) and social functioning (p=0.036) scores than MRM group. Only fatigue under symptom scales scored significantly lower in favour of BCS over the MRM group (p=0.010). The other domain score differences were not statistically different. There are inconsistent, conflicting and some convergent findings in other parts of the world as concerns QOL comparison of BCS and MRM. However, these were not specific to EBC. The findings in our study are similar to those by Yasemin Z *et al*. and Acil *et al*. Both of these studies were conducted in Turkey utilising the EORTC QLQ C30 as one of the questionnaires. They found the QOL scores were better in the BCT group as compared to the MRM group. In the later study, they also found better functional scores and symptom scores in the BCS arm better than the MRM group [23,28]. In contrast, Deepa KV *et al*. in India found a better QOL of patients who received MRM as compared to BCS in the initial 5 years, however, an equated QOL after 5 years was seen [29]. Chin Chong Huang *et al*. found far contrasting conclusions that MRM offered a better QOL than the BCS among Taiwanese women post adjuvant therapy [17]. Majewski J *et al*. in a review study involving eight randomised controlled trials that compared QOL scores of patients after BCS to MRM found that four of the studies reported a negative impact of MRM as compared to BCS while the other four found no difference. Mastectomy had the most negative impact on the body image and bio-psychological aspect of QOL [30]. The inconsistency in the QOL scores in all the above studies points to the fact that QOL perception may vary from place to place and may be influenced by socioeconomic factors and cultural connotations.

Physical functioning is the ability to perform basic routine and daily living tasks while social functioning is how one interacts with the environment and other people in the society. These were found to be significantly poor among the MRM group. This could be explained by the post-surgical complications associated with MRM including lymphedema and arm stiffness which may invariably affect the ability to perform hand functions as per a study by Erickson VS *et al*. [31]. Also, body image has been seen to deteriorate among patients after MRM [30]. This will eventually affect their social interactions. This may also be related to socio-cultural nuances and stigma around mastectomy.

### Factors affecting QOL of patients one year after surgical management for early breast cancer

Multivariate analysis revealed significant impact of comorbidities, time from surgery and level of education on the QOL of patients after surgery for EBC (Table 5). Being five years out of surgery (β=-12.329, CI -24.265 - -2.373, p= 0.022) was associated with a worse QOL as compared to those one-year post-surgery. This contrasts from findings by Deepa KV *et al*. who found a statistically similar QOL score in both MRM and BCS at 5 years after surgery [29]. Noteworthy is Dano D *et al*. who found that the general QOL of patients improved over time after initiating chemotherapy which was seen to be detrimental to the QOL [32]. This improvement in QOL was partly attributed to a strong family and social support system [32]. This deterioration in our patient study population may then be explained by a poor support system as seen by a poor attendance of the survivors meeting in the study facility (AKUHN). Support systems have been seen to help cancer patients cope with distress after and during treatment [33].

In our study, a higher level of education (ß=31.85, CI 16.913 - 51.139, p=0.000) was found to favour a better QOL after MRM or BCT. Several studies have had similar findings [23,34]. Acil *et al*. found high school and tertiary level of education was associated with lower symptom scores and higher functional score and hence better QOL [23]. Also, Dewalt *et al*. affirmed in a systematic review that literacy level was a determinant of health outcomes and that low levels of literacy were associated with several adverse health outcomes [35]. In our study, comorbidities like DM (ß=-54.578, CI -85.679 - -34.726, p=0.000) were a significant contributor to the QOL of patients one year after surgical management of EBC. Similar findings were seen by Fu *et al*. in a study on comorbidities and QOL of breast cancer survivors [36]. There are no studies that looked at comorbidities as confounders of QOL of patients after surgical management of EBC.

**Limitations:** this study focused on; among others, symptoms resulting from the general treatment but not specifically the surgery. Other effects resulting from surgery like changes body image, sexuality, anxiety about the diagnosis and fear of recurrence would add more QOL information when using a breast-specific questionnaire. For this study to be generalised to the catchment population, a larger sample size with prospective pre-surgical and post-surgical questionnaires should be considered to track the changes in QOL resulting from the surgery. The use of old medical records has the potential of including inaccurate, incomplete entries that may alter the results. It´s important to note that surgical techniques have remained relatively unchanged over time. However, the adjuvant treatment has improved for the better with less side effects. Patients receiving older adjuvant regimens could have experienced more symptoms as compared to those on newer regimens. These are the minority in this study period.

## Conclusion

As per this study, BCS/BCT offers a better QOL compared to MRM for EBC after one year from the time of surgery at a single centre in East Africa. QOL of the patients was significantly affected by diabetes mellitus as a comorbidity. Lower levels of education and being five years out of surgery also affected the quality of life suggesting that more studies need to be done to understand the drivers behind these phenomenon.

### What is known about this topic

- *BCT and MRM have comparable clinical outcomes in terms of recurrence and overall survival in EBC;*
- *QOL assessment as a patient report outcome is essential for holistic management of cancer patients;*
- *There are contrasting findings in western and Asian data on QOL after BCT/BCS versus MRM for EBC and no data in African setting*.

### What this study adds

- *BCT offers a better QOL, symptom and functional score as compared to MRM for EBC in this East African region;*
- *Being five years after surgical treatment, comorbidities like diabetes and lower level of education of patients negatively affect QOL after surgery for EBC*.

## Annexes

**Annex 1**: english EORTC QLQ-C30 (version 3) (PDF-50KB)

**Annex 2**: kiswahili EORTC QLQ-C30 (PDF 151KB)

**Annex 3**: data collection sheet (PDF 26KB)

## References

[1] Sung H, Ferlay J, Siegel RL, Laversanne M, Soerjomataram I, Jemal A (2021). Global Cancer Statistics 2020: GLOBOCAN Estimates of Incidence and Mortality Worldwide for 36 Cancers in 185 Countries. CA: a cancer journal for clinicians.

[2] Sawe RT, Kerper M, Badve S, Li J, Sandoval-Cooper M, Xie J (2016). Aggressive breast cancer in western Kenya has early onset, high proliferation, and immune cell infiltration. BMC cancer.

[3] Fregene A, Newman LA (2005). Breast cancer in sub-Saharan Africa: How does it relate to breast cancer in African-American women?. Cancer 2005.

[4] Corbex M, Bouzbid S, Boffetta P (2014). Features of breast cancer in developing countries, examples from North-Africa. European Journal of Cancer.

[5] Othieno-Abinya NA, Musibi A, Nyongesa C, Omollo R, Njihia B, Nyawira B (2018). Report on breast cancer care (BRECC) registry at the Kenyatta National Hospital, Nairobi, Kenya. Journal of Clinical Oncology.

[6] Makanga W WR, Saidi HA (2013). Profile of Female Breast Cancer Patients in a Kenyan Urban Private Hospital. The Annals of African Surgery.

[7] Weber WP, Soysal SD, Zeindler J, Kappos EA, Babst D, Schwab F (2017). Current standards in oncoplastic breast conserving surgery. Breast (Edinburgh Scotland).

[8] Fisher B, Anderson S, Redmond CK, Wolmark N, Wickerham DL, Cronin WM (1995). Reanalysis and Results after 12 Years of Follow-up in a Randomized Clinical Trial Comparing Total Mastectomy with Lumpectomy with or without Irradiation in the Treatment of Breast Cancer. New England Journal of Medicine.

[9] Fisher B, Anderson S, Bryant J, Margolese RG, Deutsch M, Fisher ER (2002). Twenty-year follow-up of a randomized trial comparing total mastectomy, lumpectomy, and lumpectomy plus irradiation for the treatment of invasive breast cancer. The New England journal of medicine.

[10] Veronesi U, Salvadori B, Luini A, Greco M, Saccozzi R, del Vecchio M (1995). Breast conservation is a safe method in patients with small cancer of the breast: long-term results of three randomised trials on 1,973 patients. European journal of cancer (Oxford, England: 1990).

[11] Veronesi U, Cascinelli N, Mariani L, Greco M, Saccozzi R, Luini A (2002). Twenty-year follow-up of a randomized study comparing breast-conserving surgery with radical mastectomy for early breast cancer. The New England journal of medicine.

[12] van Dongen JA, Voogd AC, Fentiman IS, Legrand C, Sylvester RJ, Tong D (2000). Long-term results of a randomized trial comparing breast-conserving therapy with mastectomy: European Organization for Research and Treatment of Cancer 10801 trial. Journal of the National Cancer Institute.

[13] Jacobson JA, Danforth DN, Cowan KH, d'Angelo T, Steinberg SM, Pierce L (1995). Ten-year results of a comparison of conservation with mastectomy in the treatment of stage I and II breast cancer. The New England journal of medicine.

[14] Poggi MM, Danforth DN, Sciuto LC, Smith SL, Steinberg SM, Liewehr DJ (2003). Eighteen-year results in the treatment of early breast carcinoma with mastectomy versus breast conservation therapy: the National Cancer Institute Randomized Trial. Cancer.

[15] Montazeri A (2009). Quality of life data as prognostic indicators of survival in cancer patients: an overview of the literature from 1982 to 2008. Health and quality of life outcomes.

[16] Gavric Z, Vukovic-Kostic Z (2015). Assessment of Quality of Life of Women with Breast Cancer. Global journal of health science.

[17] Huang C-C, Lien H-H, Tu S-H, Huang C-S, Jeng J-Y, Chao H-L (2010). Quality of life in Taiwanese breast cancer survivors with breast-conserving therapy. J Formos Med Assoc.

[18] Tripathi L, Datta S, Agrawal S, Chatterjee S, Ahmed R (2017). Stigma perceived by women following surgery for breast cancer. Indian Journal of Medical and Paediatric Oncology.

[19] Akezaki Y, Nakata E, Kikuuchi M, Tominaga R, Kurokawa H, Okamoto M (2021). Investigation of Factors Affecting Early Quality of Life of Patients after Breast Cancer Surgery. Healthcare.

[20] Osoba D, Rodrigues G, Myles J, Zee B, Pater J (1998). Interpreting the significance of changes in health-related quality-of-life scores. Journal of Clinical Oncology.

[21] Fayers PM AN, Bjordal K, Groenvold M, Curran D, Bottomley A, on, Group botEQoL (2001). The EORTC QLQ-C30 Scoring Manual.

[22] Glueck DHC S-C, Shao J, Wang H (2008). Sample Size Calculations in Clinical Research. Biometrics.

[23] Acil H, Cavdar I (2014). Comparison of quality of life of Turkish breast cancer patients receiving breast conserving surgery or modified radical mastectomy. Asian Pac J Cancer Prev.

[24] Bulotiene G, Veseliunas J, Ostapenko V (2007). Quality of life of Lithuanian women with early stage breast cancer. BMC Public Health.

[25] Elham Bahrami KCAaMAB (2017). The Effect of Surgery Type on the Quality of Life in Breast Cancer Patients: A Mini Review. Crimsom publishers; investigation in gynecology research and women health.

[26] Agency IN (2020). The world fact book 2020. The world fact book.

[27] Vanderpuye V, Grover S, Hammad N, Pooja Prabhakar, Simonds H, Olopade F (2017). An update on the management of breast cancer in Africa. Infectious Agents and Cancer.

[28] Yasemin Z KA, Serhat G, Atilla Ç Ercüment T (2009). Effects of breast conserving surgery in quality of life in breast cancer patients. The Journal of Breast Health.

[29] Deepa KV, Gadgil A, Löfgren J, Mehare S, Bhandarkar P, Roy N (2020). Is quality of life after mastectomy comparable to that after breast conservation surgery? A 5-year follow up study from Mumbai. India, Quality of Life Research.

[30] Majewski JM, Lopes ADF, Davoglio T, Leite JCdC (2012). Quality of life of women recovering from breast cancer after being subjected to mastectomies compared with those who had conservative surgery: a review of the literature. Cien Saude Colet.

[31] Erickson VS, Pearson ML, Ganz PA, Adams J, Kahn KL (2001). Arm Edema in Breast Cancer Patients. JNCI: Journal of the National Cancer Institute.

[32] Dano D, Hénon C, Sarr O, Ka K, Ba M, Badiane A (2019). Quality of Life During Chemotherapy for Breast Cancer in a West African Population in Dakar, Senegal: A Prospective Study. Journal of Global Oncology.

[33] Weis J (2003). Support groups for cancer patients. Support Care Cancer.

[34] Janz NK, Mujahid M, Lantz PM, Fagerlin A, Salem B, Morrow M (2005). Population-based study of the relationship of treatment and sociodemographics on quality of life for early stage breast cancer. Quality of Life Research.

[35] Dewalt DA, Berkman ND, Sheridan S, Lohr KN, Pignone MP (2004). Literacy and health outcomes: a systematic review of the literature. J Gen Intern Med.

[36] Fu MR, Axelrod D, Guth AA, Cleland CM, Ryan CE, Weaver KR (2015). Comorbidities and Quality of Life among Breast Cancer Survivors: A Prospective Study. J Pers Med.

